# Editorial: Use of cannabis derivatives in veterinary medicine

**DOI:** 10.3389/fvets.2025.1539422

**Published:** 2025-03-07

**Authors:** Robin Temmerman

**Affiliations:** European College of Veterinary Pharmacology and Toxicology (ECVPT), Ghent, Belgium

**Keywords:** cannabinoids, cannabidiol (CBD), dog, cat, horse, epilepsy, osteoarthritis, pain

## Introduction

*Cannabis* species (*Cannabis* spp.) are pharmacologically diverse plants containing myriad distinct compounds, with the phytocannabinoids (pCBs) tetrahydrocannabinolic acid (THCA) and cannabidiolic acid (CBDA) and their derivatives Δ9-tetrahydrocannabinol (THC) and cannabidiol (CBD) as prime examples.

The main pharmacodynamic target of these compounds is the endocannabinoid system (ECS), which comprises of endocannabinoids (eCBs) such as anandamide (AEA) and 2-Arachidonoylglycerol (2-AG), cannabinoid receptors (CB) 1 and 2 and their anabolic/catabolic enzymes (Di Salvo et al.) (1–3). Inclusion of several eCB-like lipid mediators, their metabolic enzymes and their molecular targets forms the endocannabinoidome (2).

In human medicine, the use of cannabis-derived products is increasing globally for a variety of indications, such as post-injury and back pain, chronic and neuropathic pain, sleeping disorders, multiple sclerosis, epilepsy and others (Klatzkow et al.) (4, 5). Veterinary medicine is dovetailing this trend with growing interest from clients and veterinarians for treating medical conditions in animals with these molecules. In general, CBD is the primary entity of interest in veterinary medicine, but other molecules are being investigated, as exemplified in this Research Topic (e.g. studies with CBDA) (Klatzkow et al.; Johns et al.; Thomson et al.).

While cannabinoids show considerable veterinary therapeutic potential in the management of osteoarthritis, epilepsy, pain and other conditions, there is currently a paucity in adequately controlled studies and data to confirm the safe and effective use in these indications. Considering the current knowledge and research gap, the goal of this Research Topic was to consolidate recent findings and results of high-quality research on the pharmacokinetics and the safety and efficacy of cannabis-derivatives in animal species. This in turn will serve as a basis for further discussions and investigations in this growing therapeutic area. This editorial synthesizes findings from 16 recent studies, published in this Research Topic, highlighting the diverse applications and potential benefits of CBD and cannabis derivatives in veterinary practice.

## Pharmacokinetics and bioavailability

Understanding the pharmacokinetics (PK) of CBD and other cannabinoids is crucial for determining appropriate dosing regimens and ensuring therapeutic efficacy. Several studies in this Research Topic have focused on the pharmacokinetics of CBD in different formulations and species:

- Dogs: research comparing four CBD preparations (three liquid ones: oil-based, nanoemulsion-based and water-soluble and semi-solid formulation) in dogs revealed that liquid forms provided higher bioavailability and faster absorption compared to semi-solid forms (Limsuwan et al.). The concentration-time profiles of CBD were comparable between the oil- and water-based formulations. The CBD in all preparations reached the maximum plasma concentration within 3 h post-dose, with an average range of 92–314 μg/L, which aligns well with other Cmax values reported in CBD PK studies in dogs using same or similar dose levels (Di Salvo et al.).
  Another study demonstrated that there were no difference in PK parameters of CBD when administered either orally or transmucosally, indicating that CBD is not readily adsorbed by the oral mucosa and that CBD is probably swallowed and absorbed in the gastrointestinal tract (della Rocca et al.).
  In addition, subcutaneous injection of a liposomal-CBD formulation produced detectable CBD plasma concentrations for 6 weeks and the following PK parameters (median and range): *C*_max_ 45.2 (17.8–72.5) ng/ml, *T*_max_ 4 (2–14) days and half-life12.4 (7.7–42.6) days (Shilo-Benjamini et al.). The long-acting properties of the formulation could offer an advantage for patient and owner compliance. Finally, the study of Corsato Alvarenga et al. shows that long-term supplementation of a broad-spectrum hemp oil leads to dose-proportional accumulation in the canine body.
- Cats: a study on the oral administration of a 1:20 THC:CBD cannabis herbal extract in cats has shown that CBD and THC are quickly absorbed, with peak plasma concentrations occurring within 2–3 h post-dose (Lyons et al.). The plasma concentrations also increased dose-proportionally. Importantly, the bioavailability of CBD in cats appears to be lower than in dogs administered the same extract (6), suggesting potentially species-specific differences in absorption and metabolism. Another explanation could be the more general issue of the difficulty of oral administration to cats (Lyons et al.).
- Horses: research on horses by Thomson et al. using a cross-over design and nasogastric tube dosing (2 mg/kg and 8 mg/kg) with a CBD/CBDA-rich hemp oil product indicated that CBD levels were lower than CBDA and therefore that CBDA showed a higher bioavailability. Additionally, it was shown that CBDA was absorbed with a biphasic pattern. Reported Cmax values were: CBD and CBDA 2 mg/kg-−5.2 and 36.95 ng/mL; CBD and CBDA 8 mg/kg-−40.35 and 353.56 ng/mL. The elimination half-life of CBD and CBDA in horses was found to be relatively short or not possible to calculate due to lack of quantifiable timepoints, suggesting the need for frequent dosing to maintain therapeutic levels. Also, the product did not appear to impact the horses on neurological, behavioral and gastrointestinal levels (see below). A study from Eichler, Poźniak, et al. reported a mean Cmax of 12.17 ng/ml when CBD was dosed at 3 mg/kg. In that study, leveraging PK data from a single dose escalation study and a multiple dosing study (also discussed below), a 3-compartmental population pharmacokinetic (popPK) was built to describe and predict CBD and metabolite concentration-time profiles. Also, urine samples were analyzed, with higher CBD and metabolite concentrations (7-OH-CBD) compared to plasma. The study showed CBD is extensively metabolized and showed high volumes of tissue distribution (not corrected for bioavailability) with a resulting extended elimination phase (Eichler, Poźniak, et al.).
- Cynomolgus macaques (nonhuman primate): a study by Johns et al. investigated the PK of a CBD/CBDA-rich hemp oil (CBD/ArHO), orally administered at two dose levels (4 and 8 mg/kg) in cynomolgus macaques, a species of nonhuman primates. Mean Cmax of CBDA was around 30 times higher than CBD (456.75 ng/ml vs. 15.98 ng/ml). The PK data suggests that once daily dosing and the chosen dosing levels are insufficient in maintaining serum CBD concentrations (Johns et al.).

## Therapeutic applications

CBD has shown promise in managing various conditions in veterinary patients, including pain, inflammation, epilepsy, and anxiety, although the strength of the scientific evidence for these indications can fluctuate:

- Pain and Inflammation: the study from Klatzkow et al. investigated the therapeutic efficacy on post-operative pain following tibial plateau leveling osteotomy (TPLO) of a CBD/CBDA product (dosed at 2–2.5 mg/kg PO every 12 h for 4 weeks) in a randomized, placebo controlled, blinded clinical trial with client-owned dogs. Variables investigated included serum biochemistry, standardized veterinary assessments for pain score, weight-bearing, and lameness and the Canine Brief Pain Inventory. The study did not show a significant impact on pain or early bone healing. CBA/CBDA hemp extract administration was associated with an increase in alkaline phosphatase (ALP) and a decrease in eosinophils (see also below). Finally, there was a potential association of CBD/CBDA and reduced post-operative anxiety. The study of Shilo-Benjamini et al. has demonstrated the efficacy of a long-acting (liposomal) CBD formulation in alleviating pain and improving the quality of life in dogs with osteoarthritis with minimal adverse events. However, the results should be interpreted cautiously due to non-blinding and lack of placebo.
- Epilepsy: clinical trials have shown that CBD can reduce the frequency and severity of seizures in dogs with intractable idiopathic epilepsy (Di Salvo et al.). These findings suggest that CBD could be a valuable adjunctive treatment for epilepsy in veterinary patients.
- Dermatological conditions: a case-report in a 2-year old mixed breed dog has show therapeutic efficacy of a CBD-rich full spectrum Cannabis oil for the management of the autoimmune skin disorder discoid lupus erythematosus (DLE) (da Silva et al.). CBD has also been used to manage pruritus and atopic dermatitis in dogs. The study of Mariga et al. evaluated the effectiveness of a full-spectrum high cannabidiol oil in canine atopic dermatitis (CAD) compared to a negative control (olive oil) based on the degree of pruritus, dermatological evaluation (CADESI-4 scoring) and skin histopathology. The study however could not show a therapeutic advantage of the CBD oil compared to the olive oil group (Mariga et al.).
- Behavioral disorders: CBD has been explored as a treatment for anxiety and stress-related behaviors in dogs, with some studies indicating positive outcomes (Di Salvo et al.).

## Safety and tolerability

The safety profile of CBD is a critical consideration for its use in veterinary medicine. Most studies have reported that CBD is well-tolerated in animals, with few adverse effects:

- Dogs: CBD/CBDA administered for the management of post-operative pain following TPLO did result in increased blood levels of ALP and a decrease in eosinophils and warrants caution (Klatzkow et al.). Another study reported no serious adverse events following single-dose administration of various CBD formulations (Limsuwan et al.). The study of Bookout et al. investigated the general safety of different cannabinoids in healthy beagle dogs in a randomized, non-blinded, negatively-controlled, parallel design 90-day repeat dose study with an additional 14-day recovery period. The authors report no somnolence, adverse events (AE) or serious adverse events (SAE). There were some significant changes in clinical pathology parameters (e.g. ALT, ALP and GGT), but were not considered clinically relevant. It is noted that the study from Klatzow et al. also reported an increase in ALP. The authors also highlight the low AE and SAE incidence in the US National Animal Supplement Council (NASC) Adverse Event Reporting System (NAERS).
- Cats: no significant adverse effects were observed in cats following single-dose administration of a 1:20 THC:CBD cannabis herbal extract with dose levels 2 and 5 mg/kg CBD (Lyons et al.). Coltherd et al. conducted a long-term (6 month) tolerance study with daily dosing (4 mg/kg) of a THC-free, CBD distillate in healthy cats with a negative (placebo) control group. The product was well tolerated and no clinically significant differences were found between biochemistry and hematology data.
- Horses: the study of Thomson et al. has shown that single-dose administration of CBD/CBDA-rich hemp extract is well-tolerated in horses, with no significant neurologic, behavioral, or gastrointestinal effects. Moreover, Eichler, Ehrle, Machnik, et al. and Eichler, Ehrle, Jensen, et al. found that a CBD paste administered orally (TAMACAN XL 55%^®^) was well tolerated and adverse events-free. However, both the conducted single dose escalation study (0.2, 1.0 and 3.0 mg/kg) and the multiple dosing study (CBD paste every 12 h for 15 days) did not significantly impact parameters such as heart rate, sedation level, behavioral observations or morning blood cortisol levels in healthy horses when compared to placebo.

## Future directions

While the current body of research, and this Research Topic specifically, provides valuable insights into the pharmacokinetics, safety and efficacy of CBD and other cannabinoids in veterinary medicine, several areas warrant further investigation:

- Long-term safety: more studies are needed to assess the long-term safety and potential cumulative effects of cannabinoids in various animal species and several dose levels.
- Dosing regimen and formulation optimization: future research should focus on optimizing dosing regimens (posology and duration) and formulations to maximize exposure and subsequently therapeutic benefits while minimizing adverse effects.
- Mechanisms of action: understanding the pharmacodynamics and mode of action by which cannabinoids exert their therapeutic and other (off-target) effects will help in developing targeted treatments for specific conditions.
- Comparative studies: comparative studies across different species and formulations will provide a clearer understanding of the interspecies differences inherent to veterinary medicine and the underlying physiological mechanisms in cannabinoid pharmacokinetics, safety and efficacy.

In conclusion, a growing body of evidence highlights the potential of cannabinoids as a versatile therapeutic agent in veterinary medicine, although not all claimed indications are supported robustly and the PK is showing high intra- and interspecies variability. Subsequently, the medicalization of cannabinoids presents several opportunities as well as challenges for veterinary medical professionals, making continued research essential to fully elucidate its benefits in adequately supported indications and to ensure its safe and effective use in animal health care.
